# Supplementary material for: Identification and functional analysis of non-coding regulatory small RNA FenSr3 in Bacillus amyloliquefaciens LPB-18
Source: PeerJ. 2023 May 15;11:e15236. doi: 10.7717/peerj.15236 (PMC10194069; doi:10.7717/peerj.15236)
Supplement: Supplemental Information 4 [file peerj-11-15236-s004.zip › KO/CK-vs-T1_map/map05111.html]

KEGG PATHWAY: Biofilm formation - Vibrio cholerae - Reference pathway


|  |  |
| --- | --- |
| **Biofilm formation - Vibrio cholerae - Reference pathway** |  |

[
Pathway menu
| Organism menu
| Pathway entry
| Show description
| User data mapping
]

Surface colonization and subsequent biofilm formation and development provide numerous advantages to microorganisms. On the other hand, biofilm formation is an energetically costly process and therefore must be tightly regulated and plastic, enabling biofilm bacteria to be responsive to the various environmental cues. The quorum sensing (QS) pathway of Vibrio cholerae activates the expression of VpsR, VpsT and AphA (the main activators of biofilm formation) at low cell density and HapR (the main repressor) at high cell density. At low cell density, biofilm genes, including Vibrio polysaccharide (VPS) biosynthesis genes and the major extracellular matrix genes RbmA, RbmC, and Bap1, are expressed. The QS pathway also leads to induction of virulence factors such as toxin coregulated pilus (TCP) and cholera toxin (CTX), essential for colonization of the host and enterotoxicity, respectively. The VarS/VarA signaling system responds to an unknown environmental cue and represses biofilm production by process which involves the regulatory sRNAs CsrB, CsrC and CsrD and the RNA-binding protein CsrA, interfering with LuxO of the QS pathway. The second messenger cAMP acts as a repressor of biofilm formation. cAMP is generated by adenylate cyclase CyaA, and in complex with cAMP receptor protein (CRP), this nucleotide has been shown to upregulate HapR production through the positive regulation of the CAI-I synthase CqsA and the negative regulation of Fis. The second messenger c-di-GMP is a key signalling molecule controlling motility and biofilm matrix production. High cellular levels of c-di-GMP promote enhanced transcription of genes involved in biofilm formation, possibly by promoting VpsT-mediated transcriptional activation of vps genes.

|  |  |  |
| --- | --- | --- |
| Reference pathway | 184% 150% 122% 100% 82% 67% 55% | 图片下载 |
